# The effects of the angle between the sole and the heel of heeled footwear on single and double support time, stride duration and toe off plantar flexion of females

**DOI:** 10.1186/1757-1146-7-S1-A14

**Published:** 2014-04-08

**Authors:** SAR Darshika, TDMSB Dassanayake

**Affiliations:** 1 Allied Health Sciences Unit, Faculty of Medicine, University of Colombo, Sri Lanka

## Introduction

This study was conducted to analyze the changes of gait parameters between two different footwear size groups due to change of angle between heel and sole of the footwear by keeping the same heel height for both groups.

## Study design

A cross-sectional descriptive study design.

## Research problem

The effects of the angle between the sole and the heel of heeled footwear on single and double support time, stride duration and toe off plantar flexion of females.

## Methodology

Hundred (100) female subjects were participated for the study and fifty one (51) subjects were included to the footwear size 36 group with 23.8years mean age (*SD* = 2.5) and forty nine subjects to the footwear size 40 group with 24.4 (*SD* = 2.4) years mean age. Both groups were given similar type three centimeters height heeled footwear and there was 6 greater angle locate between heel and sole of size 36 footwear than size 40.Two cameras (frontal stationary camera and lateral running camera) were used to capture the walking trials and each individual perform three trials. Captured two dimensional walking trials were analyzed with Motion view 8.0 video analysis software. Independent sample t-test in Statistical Package for Social Sciences Statistics version 17.0 (SPSS Statistics 17.0) was used to analyze the data.

## Results

Mean toe off phase plantar flexion and double support time was shown a significant difference (*p*<*0.05*) while the single support time and stride duration was not shown a significance (*p*>*0.05*).

## Conclusion

The angle between heal and sole of footwear significantly deviate gait parameters so rather than heel height footwear type it need to consider about shoe length.

